# Association between *LAG3/CD4* Genes Variants and Risk for Multiple Sclerosis

**DOI:** 10.3390/ijms232315244

**Published:** 2022-12-03

**Authors:** Elena García-Martín, José A. G. Agúndez, Javier Gómez-Tabales, Julián Benito-León, Jorge Millán-Pascual, María Díaz-Sánchez, Patricia Calleja, Laura Turpín-Fenoll, Hortensia Alonso-Navarro, Esteban García-Albea, José Francisco Plaza-Nieto, Felix Javier Jiménez-Jiménez

**Affiliations:** 1 University Institute of Molecular Pathology Biomarkers, Universidad de Extremadura, ARADyAL Instituto de Salud Carlos III, E10003 Cáceres, Spain; 2 CIBERNED, Centro de Investigación Biomédica en Red de Enfermedades Neurodegenerativas, Instituto de Salud Carlos III, 28031 Madrid, Spain; 3 Service of Neurology, Hospital Universitario Doce de Octubre, 28041 Madrid, Spain; 4 Section of Neurology, Hospital La Mancha-Centro, E13600 Ciudad Real, Spain; 5 Section of Neurology, Hospital Universitario del Sureste, E28500 Madrid, Spain; 6 Department of Medicine-Neurology, Hospital “Príncipe de Asturias”, Universidad de Alcalá, 28805 Madrid, Spain

**Keywords:** multiple sclerosis, genetics, genetic polymorphisms, LAG3 gene, CD4 gene, risk factors

## Abstract

Several recent works have raised the possibility of the contribution of the lymphocyte activation gene 3 (LAG3) protein in the inflammatory processes of multiple sclerosis (MS). Results of studies on the possible association between *LAG3* gene variants and the risk of MS have been inconclusive. In this study, we tried to show the possible association between the most common single nucleotide variants (SNVs) in the *CD4* and *LAG3* genes (these two genes are closely related) and the risk of MS in the Caucasian Spanish population. We studied the genotypes and allelic variants *CD4 rs1922452*, *CD4 rs951818*, and *LAG3 rs870849* in 300 patients diagnosed with MS and 400 healthy patients using specific *TaqMan*-based qPCR assays. We analyzed the possible influence of the genotype frequency on age at the onset of MS, the severity of MS, clinical evolutive subtypes of MS, and the HLADRB1*1501 genotype. The frequencies of the *CD4 rs1922452*, *CD4 rs951818*, and *LAG3 rs870849* genotypes and allelic variants were not associated with the risk of MS and were unrelated to gender, age at onset and severity of MS, the clinical subtype of MS, and HLADRB1*1501 genotype. The results of the current study showed a lack of association between the *CD4 rs1922452*, *CD4 rs951818*, and *LAG3 rs870849* SNVs and the risk of developing MS in the Caucasian Spanish population.

## 1. Introduction

Multiple sclerosis (MS) is a chronic autoimmune disease with a genetic predisposition, and affects the central nervous system, which is mainly characterized by inflammation, demyelination, and neuronal degeneration. Genome-wide association studies (GWAS) have confirmed the association of more than 200 loci of genome-wide significance with the risk of developing MS, which can explain almost half of its heritability [1,2], but for most of them, the odds ratio (OR) is somewhat modest (1.05 to 1.20), and only *HLA* (in particular the HLA-DRB1*15:01 haplotype) has shown a strong association with MS risk [1]. Together with the likely contribution of genetic predisposition to the etiopathogenesis of MS, it has been suggested that there is a possible role played by some environmental factors (causing gene-environment and environment-environment interactions), including pro-inflammatory states, such as infectious exposures (mainly Epstein-Barr virus seropositivity or exposure), smoking, obesity, and low sun exposure/low vitamin D levels) in MS onset and progression [3,4,5].

LAG3 protein is encoded by the *lymphocyte activation gene 3* (*LAG3*), also known as *CD223* gene (chromosome 12p13.31; MIM 153337, gene ID 3902). This protein is expressed by regulatory T cells, both activated and exhausted CD4+ and CD8+ T cells, and microglia, and its main mechanism of action is to deliver inhibitory signals that are involved in the regulation of immune cell homeostasis, T cell activation and proliferation, production of cytokines, cytolytic activity, and other functions related to inflammatory responses [6,7]. The *CD4 molecule* gene, (*CD4*; MIM 186940; gene ID 920), which is closely related to the *LAG3* gene, encodes the CD4 membrane glycoprotein of T lymphocytes. This glycoprotein is also an important mediator of immune and inflammatory responses.

Several recent studies have shown data suggesting a possible relationship between LAG3 protein and MS:

(A) Patients with untreated MS [8,9] and using disease modification therapies [6] showed lower LAG3 expression levels in peripheral blood mononuclear cells as compared with healthy controls, which were significantly higher in MS patients with low Expanded Disability Status Scale (EDSS) than in those with high EDSS [8], were correlated with the 1-year progression index [5], and were not affected by disease modification therapies [9].
(B) Decreases in the levels of LAG3 in peripheral blood mononuclear cells were age-dependent in healthy controls but not in patients with MS, and was more pronounced in young patients with primary progressive MS than in patients with relapsing-remitting MS, suggesting an accelerated aging process in patients with progressive MS [10].
(C) LAG3 gene was up-regulated by interferon-beta in lymphoblast cell lines of MS patients and primary B cells [11].
(D) The amelioration of clinical symptoms and decrease in inflammation in the myelin oligodendrocyte glycoprotein (MOG)35–55-induced mouse experimental autoimmune encephalomyelitis (EAE) by cannabidiol therapy seems to be related, among other factors, to the up-regulation of *LAG3* gene on CD4(+)CD25(−) accessory T cells leading to elevated levels of LAG3 mRNA, as was shown by studies using co-cultures of MOG35–55-activated and spleen-derived antigen-presenting cells [12,13].
(E) Environmental stimuli-induced intraepithelial CD4(+) lymphocytes in the gut epithelium or MOG(35–55)-specific receptor transgenic mice, which inhibit EAE and infiltrate the central nervous system (CNS), markedly up-regulate Lag3 expression in the CNS, inhibiting inflammation [14].
(F) CD4(+) memory T cells expressing the gut-homing chemokine receptor CCR9, which infiltrate the inflamed CNS in the EAE model of MS, express LAG3, and a high proportion of CCR9(+) cells isolated from the cerebrospinal fluid of patients with RRMS and neuromyelitis optica express LAG3 [15].

Several single nucleotide variants (SNVs) in the *LAG3* and *CD4* genes have been found to be related to the severity of primary immune thrombocytopenia (rs870849 T > C) [16], disease progression, mortality of sepsis (rs951818 C > A) [17], and risk for Parkinson’s disease [18,19]. Despite *LAG3/CD4* genes, SNVs have not been found to be associated with the risk for MS in GWAS, and taking into account the previously commented possible relationship between LAG3 protein and MS, it seems reasonable to investigate the possible association between *LAG3/CD4* SNVs in the risk for MS. This issue has been of the topic of several studies, although the results have been controversial [20,21,22,23]. The current replication study aimed to establish whether the most common SNVs in these genes showed an association with the risk of MS in the Spanish Caucasian population.

## 2. Results

The frequencies of the *CD4* rs1992452, *CD4* rs951818, and *LAG3* rs878049 genotypes and allelic variants that were in the Hardy–Weinberg equilibrium, both in MS patients and in controls, did not differ significantly between the two study groups when comparing the whole series (Table 1) and when analyzed each gender separately (Table 2).

A comparison of MS patients stratified according to the age at onset of the disease, using the median value of 31 years as the cut-off point, showed no significant differences in the distribution of genotypes and allelic variants of the three SNVs studied in patients with age at onset ≥31 than those with age at onset ≤ 30 years (Table 3). Similarly, we did not find significant differences in the distribution of genotypes and allelic variants between patients with EDSS score < 3 when compared with those with EDSS score ≥3, 3 being the median value (Table 4). To analyze whether the age at onset could be related to the EDSS score, we performed a linear regression analysis between these two parameters and did not identify a statistically significant correlation (*p* = 0.061). Additionally, we analyzed the correlation between the age and the EDSS score, and we did not identify any significant correlation (*p* = 0.624).

The distributions of *CD4* rs1992452, *CD4* rs951818, and *LAG3* rs878049 genotypes and allelic frequencies were similar between “relapsing–remitting”, “secondary progressive”, and “primary progressive” clinical evolutive subtypes of MS and controls, except for a significantly higher frequency of rs870849CC genotype and rs870849C allelic variant in patients with “relapsing–remitting” compared with those with “primary progressive” MS, which disappeared after correction for multiple comparisons (*p* = 0.019, Pc = 0.171 for genotypes, and *p* = 0.023, PC = 0.069 for alleles) (Table 5).

Finally, the distributions of *CD4* rs1992452, *CD4* rs951818, and *LAG3* rs878049 genotypes and allelic frequencies did not differ between patients with the three possible genotypes of HLADRB1*1501, except for a significantly lower frequency of the rs1922452 A allele in patients with the genotype HLADRB1*1501 T/T compared with those with the genotype HLADRB1*1501 T/A, which disappeared after correction for multiple comparisons (Table 6).

## 3. Discussion

Despite the previously mentioned studies suggesting a possible role of LAG3 in the pathogenesis of MS [8,9,10,11,12,13,14,15], the possible contribution of polymorphisms in the *LAG3/CD4* genes to the risk for MS has not been still established.

Zhang et al. [20], in a case-control association study in the Swedish population involving 672 patients with MS and 672 healthy controls, described an association of rs1922452, rs951818, and rs870849 SNVs with the risk of MS, while they reported a lack of association of rs1882545, rs2365095, rs7488113, rs1882551, rs11227, and rs25557 SNVs with this disease. Lundmark et al. [21], in a replication study involving 1720 patients with MS and 1416 controls from Denmark, Finland, and Norway (Nordic cohort), did not confirm an association of rs1922452, rs951818, and rs870849 SNVs with MS risk. In the same study, they described an association between rs3782736, rs7957426, and rs10774450 SNVs in the *CD4* gene and the risk of MS in the Swedish cohort, but these associations were not confirmed in the Nordic cohort [21].

Al-Eitan et al. [22], in a case-control association study involving 218 MS patients and 227 controls from the Jordan Arab population, described a modest association between the rs2365095 SNV and MS risk, while they found no association of rs2365095, rs1922452, rs951818, rs870849, rs1882551, and rs11227 with MS. Furthermore, they also described a lack of association of rs2365095, rs1922452, rs951818, rs870849, rs1882551, and rs11227 SNVs with MS duration, age at MS onset, EDSS score at presentation, and clinical form of MS [23].

In the current study, involving 300 MS patients and 400 from the Spanish population, we did not find any association between the rs1992452, rs951818, and rs878049 variants (the first and the second are now ascribed to the *CD4* and the latter to the *LAG3* genes) with the risk of MS, both in the whole series and when analyzing male and female genders separately. In addition, none of the studied SNV variants was related to the age at the onset of MS, the severity of MS (assessed by using the EDSS score), the clinical evolutive subtype MS, and the HLADRB1*15 status.

However, the present study shows several limitations. Firstly, the sample size, although adequate to detect ORs of 1.5, is relatively low, and might overlook modest associations. Second, because the patients diagnosed with MS included in our cohort showed different degrees of severity, the investigation of the influence of the 3 SNVs analyzed on the disease severity or disability could have been limited. Finally, we only analyzed three SNVs in the closely related *CD4* and *LAG3* genes, and this fact does not preclude the possible implication of other SNVs in the same genes. A better approach in the future should be a prospective study including the genotyping of patients with a recent diagnosis of MS and a long-term follow-up of the initial cohort to determine the final evolutive types of MS. Taking into account all these limitations, the results of the current study suggest that rs1992452, rs951818, and rs878049 variants are not associated with the modification of the risk of MS in Caucasian Spanish people.

## 4. Materials and Methods

### 4.1. Patients and Controls

The study involved 300 Caucasian Spanish individuals diagnosed with MS according to the McDonald’s criteria for definite MS [24] and who had not been diagnosed previously with other autoimmune or neurological diseases, and 400 age- and gender-matched healthy control individuals who were unrelated to patients with MS and were included in the study if none of their first-degree relatives had either autoimmune or neurological diseases. The demographic and clinical data of the MS patients and controls are summarized in Table 7. Recruitment of MS patients was carried out in the “Multiple Sclerosis Association of Madrid” (*n* = 165), Hospital La-Mancha-Centro (Alcázar de San Juan, Ciudad Real, Spain; *n* = 65), University Hospital ‘‘Doce de Octubre’’ (Madrid; *n* = 30), University Hospital ‘‘Príncipe de Asturias’’ (Alcalá de Henares, Madrid, Spain; *n* = 25), and University Hospital of Sureste (Arganda del Rey, Madrid, Spain; *n* = 15). Controls were recruited from the University Hospital of Badajoz (Badajoz, Spain).

### 4.2. Ethical Aspects

This study, which was performed following the principles of the Helsinki’s Declaration of 1975, was approved by the Ethics Committees of Clinical Investigation of the University Hospital “Príncipe de Asturias” (University of Alcalá, Alcalá de Henares, Madrid, Spain), the University Hospital of Badajoz (Badajoz, Spain). A signature indicating informed consent after a full explanation of the procedure was an obligatory requisite for inclusion. Many of these patients had participated in previous studies of genetic association with the risk of developing MS [25,26,27,28,29,30,31,32,33,34].

### 4.3. Genotyping of CD4 rs1922452, CD4 rs951818, and LAG3 rs870849 Variants

Genotyping studies were performed using genomic DNA obtained from peripheral leukocytes of venous blood samples of patients diagnosed with MS and controls. We used a real-time PCR (Applied Biosystems 7500 qPCR thermocycler, Alcobendas, Madrid, Spain) with specific custom-designed TaqMan probes (Life Technologies, Alcobendas, Madrid, Spain) was used. We analyzed three common single nucleotide variations (SNVs) that were previously analyzed in other case-control studies involving MS patients [17,18] and PD patients [15,16]; that is, one intronic and one non-coding transcript exonic SNV with high allele frequencies, and the only missense SNVs with allele frequencies over 0.01 in the population analyzed here. The SNVs we analyzed and the corresponding TaqMan tests performed were as follows: rs1922452 (C__11914936_10), rs951818 (C___8921385_10), and rs870849 (C___9797874_10).

### 4.4. Statistical Analysis

Statistical analyses were performed using the SPSS 27.0 version for Windows (SPSS Inc., Chicago, IL, USA). The online program https://ihg.helmholtz-muenchen.de/cgi-bin/hw/hwa1.pl (last accessed 20 August 2002) was used to confirm the Hardy–Weinberg equilibrium both in MS patients and in controls The intergroup comparison values (both for the whole series of patients with MS and controls and for each gender of MS patients and controls) were calculated using the chi-square test (or Fisher’s exact test where appropriate). We also calculated the 95% confidence intervals (95% CI) and the negative predictive values (NPV) [35]. The false discovery rate (FDR) [36] was used to calculate the correction for multiple comparison adjustments. The chi-square or Fisher’s exact tests were also used for the comparison between subgroups of MS patients according to the mean age at onset of MS, the severity of the disease, assessment with the EDSS, the clinical evolutive subtype of the disease, and the HLADRB1*1501 (rs3135388) genotype.

We calculated the sample size using a used a genetic model that analyzed the frequency of the lower allele with an odds ratio (OR) value = 1.5 (α = 0.05) from the minor allele frequencies found in healthy subjects. The respective statistical powers (two-tailed association), taking into account the sample size for this study, were 96.18% for rs1922452, 96.12% for rs951818, and 95.80% for rs870849 variant alleles.

## Figures and Tables

**Table 1:** Genotypes and allelic variants of patients with MS and of healthy volunteers. The values in each cell represent the number (percentage; 95% confidence intervals). NPV: negative predictive value.

	MS Patients (*n* = 300, 600 Alleles)	Controls (*n* = 400, 800 Alleles)	Intergroup Comparison OR (95% CI), *p*; Pc; NPV (95% CI)
**GENOTYPES**			
rs1922452 A/A	51 (17.0; 12.7–21.3)	67 (16.8; 13.1–20.4)	1.02 (0.68–1.52); 0.930; 0.952; 0.57 (0.56–0.59)
rs1922452 A/G	138 (46.0; 40.4–51.6)	189 (47.3; 42.4–52.1)	0.95 (0.70–1.28); 0.743; 0.952; 0.57 (0.53–0.60)
rs1922452 G/G	111 (37.0; 31.5–42.5)	144 (36.0; 31.3–40.7)	1.04 (0.77–1.43); 0.786; 0.952; 0.58 (0.55–0.60)
rs951818 A/A	103 (34.3; 29.0–39.7)	145 (36.3; 31.5–41.0)	0.92 (0.67–1.26); 0.600; 0.952; 0.56 (0.54–0.59)
rs951818 A/C	151 (50.3; 44.7–56.0)	193 (48.3; 43.4–53.1)	1.09 (0.81–1.47); 0.586; 0.952; 0.58 (0.54–0.62)
rs951818 C/C	46 (15.3; 11.3–19.4)	62 (15.5; 12.0–19.0)	0.99 (0.65–1.50); 0.952; 0.952; 0.57 (0.56–0.59)
rs870849 C/C	114 (38.0; 32.5–43.5)	158 (39.5; 34.7–44.3)	0.94 (0.69–1.28); 0.687; 0.952; 0.57 (0.54–0.60)
rs870849 C/T	145 (48.3; 42.7–54.0)	192 (48.0; 43.1–52.9)	1.01 (0.75–1.37); 0.930; 0.952; 0.57 (0.54–0.61)
rs870849 T/T	41 (13.7; 9.8–17.6)	50 (12.5; 9.3–15.7)	1.11 (0.71–1.73); 0.650; 0.952; 0.58 (0.56–0.59)
**ALLELES**			
rs1922452 A	240 (40.0; 36.1–43.9)	323 (40.4; 37.0–43.8)	0.98 (0.79–1.22); 0.887; 0.887; 0.57 (0.55–0.59)
rs1922452 G	360 (60.0; 56.1–63.9)	477 (59.6; 56.2–63.0)	1.02 (0.82–1.26); 0.887; 0.887; 0.57 (0.54–0.61)
rs951818 A	357 (59.5; 55.6–63.4)	483 (60.4; 57.0–63.8)	0.96 (0.78–1.20); 0.741; 0.887; 0.57 (0.53–0.60)
rs951818 C	243 (40.5; 36.6–44.4)	317 (39.6; 36.2–43.0)	1.04 (0.84–1.29); 0.741; 0.887; 0.58 (0.55–0.60)
rs870849 C	373 (62.2; 58.3–66.0)	508 (63.5; 60.2–66.8)	0.95 (0.76–1.18); 0.609; 0.887; 0.56 (0.53–0.60)
rs870849 T	227 (37.8; 34.0–41.7)	292 (36.5; 33.2–39.8)	1.06 (0.85–1.32); 0.609; 0.887; 0.58 (0.56–0.60)

**Table 2:** Genotypes and allelic variants of patients with MS and of healthy volunteers, distributed by gender. The values in each cell represent the number (percentage; 95% confidence intervals). NPV: negative predictive value.

	MS Women (*n* = 207, 414 Alleles)	Control Women (*n* = 276, 552 Alleles)	Intergroup Comparison OR (95% CI), *p*; Pc; NPV (95% CI)	MS Men (*n* = 93, 186 Alleles)	Control Men (*n* = 124, 248 Alleles)	Intergroup Comparison OR (95% CI), *p*; Pc; NPV (95% CI)
**GENOTYPES**						
rs1922452 A/A	35 (16.9; 11.8–22.0)	47 (17.0; 12.6–21.5)	0.99 (0.61–1.60); 0.972; 1.000; 0.57 (0.55–0.59)	16 (17.2; 9.5–24.9)	20 (16.1; 9.7–22.6)	1.08 (0.53–2.22); 0.833; 0.833; 0.58 (0.54–0.61)
rs1922452 A/G	97 (46.9; 40.1–53.7)	129 (46.7; 40.9–52.6)	1.01 (0.70–1.44); 0.979; 1.000; 0.57 (0.53–0.62)	41 (44.1; 34.0–54.2)	61 (49.2; 40.4–58.0)	0.81 (0.48–1.40); 0.457; 0.738; 0.55 (0.48–0.61)
rs1922452 G/G	75 (36.2; 29.7–42.8)	100 (36.2; 30.6–41.9)	1.00 (0.69–1.46); 1.000; 1.000; 0.57 (0.54–0.61)	36 (38.7; 28.8–48.6)	43 (34.7; 26.3–43.1)	1.19 (0.68–2.08); 0.542; 0.738; 0.59 (0.53–0.64)
rs951818 A/A	72 (34.8; 28.3–41.3)	99 (35.9; 30.2–41.5)	0.95 (0.65–1.39); 0.805; 1.000; 0.57 (0.53–0.60)	31 (33.3; 23.8–42.9)	48 (38.7; 30.1–47.3)	0.79 (0.45–1.39); 0.416; 0.738; 0.55 (0.50–0.60)
rs951818 A/C	105 (50.7; 43.9–57.5)	134 (48.6; 42.7–54.4)	1.09 (0.76–1.56); 0.637; 1.000; 0.58 (0.54–0.63)	46 (49.5; 39.3–59.6)	59 (47.6; 38.8–56.4)	1.08 (0.63–1.85); 0.784; 0.833; 0.58 (0.51–0.65)
rs951818 C/C	30 (14.5; 9.7–19.3)	43 (15.6; 11.3–19.9)	0.92 (0.55–1.52); 0.742; 1.000; 0.57 (0.55–0.59)	16 (17.2; 9.5–24.9)	17 (13.7; 7.7–19.8)	1.31 (0.62–2.75); 0.479; 0.738; 0.58 (0.55–0.61)
rs870849 C/C	80 (38.6; 32.0–45.3)	108 (39.1; 33.4–44.9)	0.98 (0.68–1.42); 0.914; 1.000; 0.57 (0.53–0.61)	34 (36.6; 26.8–46.3)	50 (40.3; 31.7–49.0)	0.85 (0.49–1.48); 0.574; 0.738; 0.56 (0.50–0.61)
rs870849 C/T	97 (46.9; 40.1–53.7)	135 (48.9; 43.0–54.8)	0.92 (0.64–1.32); 0.655; 1.000; 0.56 (0.52–0.61)	48 (51.6; 41.5–61.8)	56 (45.2; 36.4–53.9)	1.30 (0.76–2.22); 0.348; 0.738; 0.60 (0.53–0.67)
rs870849 T/T	30 (14.5; 9.7–19.3)	33 (12.0; 8.1–15.8)	1.25 (0.73–2.12); 0.413; 1.000; 0.58 (0.56–0.60)	11 (11.8; 5.3–18.4)	18 (14.5; 8.3–20.7)	0.79 (0.36–1.76); 0.566; 0.738; 0.56 (0.54–0.59)
**ALLELES**						
rs1922452 A	167 (40.3; 35.6–45.1)	223 (40.4; 36.3–44.5)	1.00 (0.77–1.29); 0.985; 1.000; 0.57 (0.55–0.60)	73 (39.2; 32.2–46.3)	101 (40.7; 34.6–46.8)	0.94 (0.64–1.39); 0.756; 0.909; 0.57 (0.53–0.61)
rs1922452 G	247 (59.7; 54.9–64.4)	329 (59.6; 55.5–63.7)	1.00 (0.77–1.30); 0.985; 1.000; 0.57 (0.53–0.61)	113 (60.8; 53.7–67.8)	147 (59.3; 53.2–65.4)	1.06 (0.72–1.57); 0.756; 0.909; 0.58 (0.52–0.64)
rs951818 A	249 (60.1; 55.4–64.9)	332 (60.1; 56.1–64.2)	1.00 (0.77–1.30); 1.000; 1.000; 0.57 (0.53–0.61)	108 (58.1; 51.0–65.2)	155 (62.5; 56.5–68.5)	0.83 (0.56–1.23); 0.305; 0.909; 0.54 (0.48–0.60)
rs951818 C	165 (39.9; 35.1–44.6)	220 (39.9; 35.8–43.9)	1.00 (0.77–1.30); 1.000; 1.000; 0.57 (0.55–0.60)	78 (41.9; 34.8–49.0)	93 (37.5; 31.5–43.5)	1.20 (0.82–1.78); 0.350; 0.909; 0.59 (0.55–0.63)
rs870849 C	257 (62.1; 57.4–66.8)	351 (63.6; 59.6–67.6)	0.94 (0.72–1.22); 0.631; 1.000; 0.56 (0.52–0.60)	116 (62.4; 55.4–69.3)	156 (62.9; 56.9–68.9)	0.98 (0.66–1.45); 0.909; 0.909; 0.57 (0.50–0.63)
rs870849 T	157 (37.9; 33.2–42.6)	201 (36.4; 32.4–40.4)	1.07 (0.82–1.39); 0.631; 1.000; 0.58 (0.55–0.60)	70 (37.6; 30.7–44.6)	92 (37.1; 31.1–43.1)	1.02 (0.69–1.52); 0.909; 0.909; 0.57 (0.54–0.61)

**Table 3:** Genotypes and allelic variants of patients with MS according to the age at onset. The values in each cell represent the number (percentage; 95% confidence intervals).

	Age at Onset ≥ 31 (*n* = 154, 308 Alleles)	Age at Onset ≤ 30 (*n* = 146, 292 Alleles)	Intergroup Comparison OR (95% CI), *p*; Pc; NPV (95% CI)
**GENOTYPES**			
rs1922452 A/A	23 (14.9; 9.3–20.6)	28 (19.2; 12.8–25.6)	0.74 (0.40–1.36); 0.329; 0.615; 0.47 (0.45–0.50)
rs1922452 A/G	76 (49.4; 41.5–57.2)	62 (42.5; 34.4–50.5)	1.32 (0.84–2.08); 0.233; 0.615; 0.52 (0.46–0.57)
rs1922452 G/G	55 (35.7; 28.1–43.3)	56 (38.4; 30.5–46.2)	0.89 (0.56–1.43); 0.636; 0.818; 0.48 (0.42–0.57)
rs951818 A/A	47 (30.5; 23.2–37.8)	56 (38.4; 30.5–46.2)	0.71 (0.44–1.14); 0.154; 0.615; 0.46 (0.41–0.50)
rs951818 A/C	78 (50.6; 42.8–58.5)	73 (50.0; 41.9–58.1)	1.03 (0.65–1.61); 0.911; 0.988; 0.49 (0.43–0.55)
rs951818 C/C	29 (18.8; 12.7–25.0)	17 (11.6; 6.4–16.8)	1.76 (0.92–3.36); 0.085; 0.615; 0.51 (0.48–0.53)
0.51 (0.48−0.53)	55 (35.7; 28.1−43.3)	59 (40.4; 32.5−48.4)	0.82 (0.51−1.31); 0.403; 0.615; 0.47 (0.42−0.52)
rs870849 C/T	78 (50.6; 42.8−58.5)	67 (45.9; 37.8−54.0)	1.21 (0.77−1.91); 0.410; 0.615; 0.51 (0.45−0.57)
rs870849 T/T	21 (13.6; 8.2–19.1)	20 (13.7; 8.1–19.3)	1.00 (0.52–1.92); 0.988; 0.988; 0.49 (0.46–0.51)
**ALLELES**			
rs1922452 A	122 (39.6; 34.1–45.1)	118 (40.4; 34.8–46.0)	0.97 (0.70–1.34); 0.842; 0.842; 0.48 (0.45–0.52)
rs1922452 G	186 (60.4; 54.9–65.9)	174 (59.6; 54.0–65.2)	1.03 (0.75–1.43); 0.842; 0.842; 0.49 (0.44–0.54)
rs951818 A	172 (55.8; 50.3–61.4)	185 (63.4; 57.8–68.9)	0.73 (0.53–1.02); 0.061; 0.183; 0.44 (0.39–0.49)
rs951818 C	136 (44.2; 38.6–49.7)	107 (36.6; 31.1–42.2)	1.37 (0.99–1.90); 0.061; 0.183; 0.52 (0.48–0.55)
rs870849 C	188 (61.0; 55.6–66.5)	185 (63.4; 57.8–68.9)	0.91 (0.65–1.26); 0.559; 0.839; 0.47 (0.42–0.53)
rs870849 T	120 (39.0; 33.5–44.4)	107 (36.6; 31.1–42.2)	1.10 (0.79–1.54); 0.559; 0.839; 0.50 (0.46–0.53)

**Table 4:** Genotypes and allelic variants of patients with MS according to the EDSS scores. The values in each cell represent the number (percentage; 95% confidence intervals).

	EDSS Score < 3 (*n* = 149, 298 Alleles)	EDSS Score ≥ 3 (*n* = 151, 302 Alleles)	Intergroup Comparison OR (95% CI), *p*; Pc; NPV (95% CI)
**GENOTYPES**			
rs1922452 A/A	26 (17.4; 11.4–23.5)	25 (16.6; 10.6–22.5)	1.07 (0.58–1.95); 0.837; 0.976; 0.51 (0.48–0.53)
rs1922452 A/G	69 (46.3; 38.3–54.3)	69 (45.7; 37.7–53.6)	1.03 (0.65–1.61); 0.915; 0.976; 0.51 (0.45–0.56)
rs1922452 G/G	54 (36.2; 28.5–44.0)	57 (37.7; 30.0–45.5)	0.94 (0.59–1.50); 0.787; 0.976; 0.50 (0.45–0.54)
rs951818 A/A	51 (34.2; 26.6–41.8)	52 (34.4; 26.9–42.0)	0.99 (0.62–1.60); 0.970; 0.976; 0.50 (0.46–0.55)
rs951818 A/C	75 (50.3; 42.3–58.4)	76 (50.3; 42.4–58.3)	1.01 (0.63–1.63); 0.976; 0.976; 0.50 (0.46–0.55)
rs951818 C/C	23 (15.4; 9.6–21.2)	23 (15.2; 9.5–21.0)	1.02 (0.54–1.90); 0.961; 0.976; 0.50 (0.48–0.53)
rs870849 C/C	55 (36.9; 29.2–44.7)	59 (39.1; 31.3–46.9)	0.91 (0.57–1.46); 0.700; 0.976; 0.50 (0.45–0.54)
rs870849 C/T	69 (46.3; 38.3–54.3)	76 (50.3; 42.4–58.3)	0.85 (0.54–1.34); 0.486; 0.976; 0.48 (0.43–0.54)
rs870849 T/T	25 (16.8; 10.8–22.8)	16 (10.6; 5.7–15.5)	1.70 (0.87–3.34); 0.120; 0.976; 0.52 (0.50–0.54)
**ALLELES**			
rs1922452 A	121 (40.6; 35.0–46.2)	119 (39.4; 33.9–44.9)	1.05 (0.76–1.46); 0.764; 0.959; 0.51 (0.47–0.54)
rs1922452 G	177 (59.4; 53.8–65.0)	183 (60.6; 55.1–66.1)	0.95 (0.69–1.32); 0.764; 0.959; 0.50 (0.45–0.55)
rs951818 A	177 (59.4; 53.8–65.0)	180 (59.6; 54.1–65.1)	0.99 (0.72–1.37); 0.959; 0.959; 0.50 (0.45–0.55)
rs951818 C	121 (40.6; 35.0–46.2)	122 (40.4; 34.9–45.9)	1.01 (0.73–1.40); 0.959; 0.959; 0.50 (0.47–0.54)
rs870849 C	179 (60.1; 54.5–65.6)	194 (64.2; 58.8–69.6)	0.84 (0.60–1.17); 0.293; 0.879; 0.48 (0.42–0.53)
rs870849 T	119 (39.9; 34.4–45.5)	108 (35.8; 30.4–41.2)	1.19 (0.86–1.66); 0.293; 0.879; 0.52 (0.49–0.55)

**Table 5:** *Genotypes* and allelic variants of patients with MS according to the evolutive type of MS. The values in each cell represent the number (percentage; 95% confidence intervals).

	Relapsing-Remitting MS *n* = 163	Secondary Progressive *n* = 94	Primary Progressive *n* = 43	Controls (*n* = 400)
**GENOTYPES**				
rs1922452 A/A	32 (19.6; 13.5–25.7)	14 (14.9; 7.7–22.1)	5 (11.6; 2.0–21.2)	67 (16.8; 13.1–20.4)
rs1922452 A/G	78 (47.9; 40.2–55.5)	41 (43.6; 33.6–53.6)	19 (44.2; 29.3–59.0)	189 (47.3; 42.4–52.1)
rs1922452 G/G	53 (32.5; 25.3–39.7)	39 (41.5; 31.5–51.4)	19 (44.2; 29.3–59.0)	144 (36.0; 31.3–40.7)
rs951818 A/A	61 (37.4; 30.0–44.9)	31 (33.0; 23.5–42.5)	11 (25.6; 12.5–38.6)	145 (36.3; 31.5–41.0)
rs951818 A/C	79 (48.5; 40.8–56.1)	47 (50.0; 39.9–60.1)	25 (58.1; 43.4–72.9)	193 (48.3; 43.4–53.1)
rs951818 C/C	23 (14.1; 8.8–19.5)	16 (17.0; 9.4–24.6)	7 (16.3; 5.2–27.3)	62 (15.5; 12.0–19.0)
rs870849 C/C	70 (42.9; 35.3–50.5)	34 (36.2; 26.5–45.9)	10 (23.3; 10.6–35.9)	158 (39.5; 34.7–44.3)
rs870849 C/T	74 (45.4; 37.8–53.0)	46 (48.9; 38.8–59.0)	25 (58.1; 43.4–72.9)	192 (48.0; 43.1–52.9)
rs870849 T/T	19 (11.7; 6.7–16.6)	14 (14.9; 7.7–22.1)	8 (18.6; 7.0–30.2)	50 (12.5; 9.3–15.7)
**ALLELES**				
rs1922452 A	142 (43.6; 38.2–48.9)	69 (36.7; 29.8–43.6)	29 (33.7; 23.7–43.7)	323 (40.4; 37.0–43.8)
rs1922452 G	184 (56.4; 51.1–61.8)	119 (63.3; 56.4–70.2)	57 (66.3; 56.3–76.3)	477 (59.6; 56.2–63.0)
rs951818 A	201 (61.7; 56.4–66.9)	109 (58.0; 50.9–65.0)	47 (54.7; 44.1–65.2)	483 (60.4; 57.0–63.8)
rs951818 C	125 (38.3; 33.1–43.6)	79 (42.0; 35.0–49.1)	39 (45.3; 34.8–55.9)	317 (39.6; 36.2–43.0)
rs870849 C	214 (65.6; 60.5–70.8)	114 (60.6; 53.7–67.6)	45 (52.3; 41.8–62.9)	508 (63.5; 60.2–66.8)
rs870849 T	112 (34.4; 29.2–39.5)	74 (39.4; 32.4–46.3)	41 (47.7; 37.1–58.2)	292 (36.5; 33.2–39.8)

Besides the comparisons shown in the table, none of the subgroups of MS patients displayed statistically significant differences between themselves or compared with control subjects.

**Table 6:** *Genotypes* and allelic variants of patients with MS distributed by HLADRB1*1501 (rs3135388) genotype. The values in each cell represent the number (percentage; 95% confidence intervals).

	HLADRB1*1501 T/T *n* = 181	HLADRB1*1501 T/A *n* = 110	Intergroup Comparison Values (T/T vs. T/A) OR (95% CI), *p*; Pc; NPV (95% CI)	HLADRB1*1501 A/A *n* = 9
**GENOTYPES**				
rs1922452 A/A	25 (13.8; 8.8–18.8)	25 (22.7; 14.9–30.6)	0.55 (0.30–1.00); 0.051; 0.392; 0.35 (0.33–0.38)	1 (11.1; −9.4–31.6)
rs1922452 A/G	82 (45.3; 38.1–52.6)	50 (45.5; 36.1–54.8)	0.99 (0.62–1.60); 0.980; 0.980; 0.38 (0.32–0.43)	6 (66.7; 35.9–97.5)
rs1922452 G/G	74 (40.9; 33.7–48.0)	35 (31.8; 23.1–40.5)	1.48 (0.90–2.44); 0.122; 0.392; 0.41 (0.37–0.46)	2 (22.2; −4.9–49.4)
rs951818 A/A	61 (33.7; 26.8–40.6)	40 (36.4; 27.4–45.4)	0.89 (0.54–1.46); 0.644; 0.828; 0.37 (0.33–0.41)	2 (22.2; −4.9–49.4)
rs951818 A/C	89 (49.2; 41.9–56.5)	58 (52.7; 43.4–62.1)	0.87 (0.54–1.39); 0.557; 0.828; 0.36 (0.30–0.42)	4 (44.4; 12.0–76.9)
rs951818 C/C	31 (17.1; 11.6–22.6)	12 (10.9; 5.1–16.7)	1.69 (0.83–3.44); 0.148; 0.392; 0.40 (0.37–0.42)	3 (33.3; 2.5–64.1)
rs870849 C/C	74 (40.9; 33.7–48.0)	37 (33.6; 24.8–42.5)	1.36 (0.83–2.24); 0.218; 0.925; 0.41 (0.36–0.45)	3 (33.3; 2.5–64.1)
rs870849 C/T	86 (47.5; 40.2–54.8)	54 (49.1; 39.7–58.4)	0.94 (0.58–1.51); 0.794; 0.893; 0.37 (0.31–0.43)	5 (55.6; 23.1–88.0)
rs870849 T/T	21 (11.6; 6.9–16.3)	19 (17.3; 10.2–24.3)	0.63 (0.32–1.23); 0.174; 0.392; 0.36 (0.34–0.39)	1 (11.1; −9.4–31.6)
**ALLELES**				
rs1922452 A	132 (36.5; 31.5–41.4)	100 (45.5; 38.9–52.0)	0.69 (0.49–0.97); 0.032; 0.096; 0.34 (0.31–0.38)	8 (44.4; 21.5–67.4)
rs1922452 G	230 (63.5; 58.6–68.5)	120 (54.5; 48.0–61.1)	1.45 (1.03–2.04); 0.032; 0.096; 0.43 (0.38–0.48)	10 (55.6; 32.6–78.5)
rs951818 A	211 (58.3; 53.2–63.4)	138 (62.7; 56.3–69.1)	0.83 (0.59–1.17); 0.290; 0.290; 0.35 (0.30–0.40)	8 (44.4; 21.5–67.4)
rs951818 C	151 (41.7; 36.6–46.8)	82 (37.3; 30.9–43.7)	1.20 (0.85–1.70); 0.290; 0.290; 0.40 (0.36–0.43)	10 (55.6; 32.6–78.5)
rs870849 C	234 (64.6; 59.7–69.6)	128 (58.2; 51.7–64.7)	1.31 (0.93–1.85); 0.119; 0.179; 0.42 (0.37–0.47)	11 (61.1; 38.6–83.6)
rs870849 T	128 (35.4; 30.4–40.3)	92 (41.8; 35.3–48.3)	0.76 (0.54–1.07); 0.119; 0.179; 0.35 (0.32–0.39)	7 (38.9; 16.4–61.4)

Besides those shown in the table, none of the subgroups of MS patients displayed statistically significant differences as compared between themselves or with control subjects. Test for trend of variant rs1922452 alleles (HLADRB1*1501 T/T vs. T/A): *p* = 0.036.

**Table 7:** Demographic and clinical data of the series studied.

Group	Controls (*n* = 400)	MS (*n* = 300)
**Age, y, mean (SD)**	44.1 (11.1)	43.9 (11.4)
**Age at onset, y, mean (SD)**	NA	32.8 (18.2)
**Female %**	276 (69.0%)	207 (69.0%)
**EDSS**	NA	3.27 (2.44)
**MS evolutive type**		
Relapsing–remitting MS	NA	163 (54.3%)
Secondary progressive MS	NA	93 (31.0%)
Primary progressive MS	NA	44 (14.7%)

EDSS expanded disability score scale.

## Data Availability

All data relating to the current study intended for reasonable use is available from J.A.G. Agúndez (University Institute of Molecular Pathology Biomarkers, University of Extremadura-UNEx, ARADyAL Instituto de Salud Carlos III, Av/de la Universidad S/N, E10071 Cáceres, Spain) and F.J. Jiménez-Jiménez (Section of Neurology, Hospital del Sureste, Arganda del Rey, Madrid, Spain).
